# The Effects of Timing of Grazing on Plant and Arthropod Communities in High-Elevation Grasslands

**DOI:** 10.1371/journal.pone.0110460

**Published:** 2014-10-22

**Authors:** Stacy C. Davis, Laura A. Burkle, Wyatt F. Cross, Kyle A. Cutting

**Affiliations:** 1 Department of Ecology, Montana State University, Bozeman, Montana, United States of America; 2 Red Rock Lakes National Wildlife Refuge, US Fish and Wildlife Service, Lima, Montana, United States of America; Dauphin Island Sea Lab, United States of America

## Abstract

Livestock grazing can be used as a key management tool for maintaining healthy ecosystems. However, the effectiveness of using grazing to modify habitat for species of conservation concern depends on how the grazing regime is implemented. Timing of grazing is one grazing regime component that is less understood than grazing intensity and grazer identity, but is predicted to have important implications for plant and higher trophic level responses. We experimentally assessed how timing of cattle grazing affected plant and arthropod communities in high-elevation grasslands of southwest Montana to better evaluate its use as a tool for multi-trophic level management. We manipulated timing of grazing, with one grazing treatment beginning in mid-June and the other in mid-July, in two experiments conducted in different grassland habitat types (i.e., wet meadow and upland) in 2011 and 2012. In the upland grassland experiment, we found that both early and late grazing treatments reduced forb biomass, whereas graminoid biomass was only reduced with late grazing. Grazing earlier in the growing season versus later did not result in greater recovery of graminoid or forb biomass as expected. In addition, the density of the most ubiquitous grassland arthropod order (Hemiptera) was reduced by both grazing treatments in upland grasslands. A comparison of end-of-season plant responses to grazing in upland versus wet meadow grasslands revealed that grazing reduced graminoid biomass in the wet meadow and forb biomass in the upland, irrespective of timing of grazing. Both grazing treatments also reduced end-of-season total arthropod and Hemiptera densities and Hemiptera biomass in both grassland habitat types. Our results indicate that both early and late season herbivory affect many plant and arthropod characteristics in a similar manner, but grazing earlier may negatively impact species of conservation concern requiring forage earlier in the growing season.

## Introduction

Grazing is a key process in grasslands that has far reaching effects on plant and animal diversity [1], [2], [3], vegetation structure [4], and ecosystem functioning [5] over multiple spatial scales. Although native ungulate grazers dominated certain landscapes prior to European settlement, many of these grasslands around the world are now grazed by domestic livestock [6]. In 2007, approximately 27% of U.S. land area was classified as grassland pasture and range for livestock production [7]. Current grazing management strategies aim to balance both ecological sustainability and economic considerations [8]. Most conservation practitioners now recognize that grazing may be used as an important land management tool [9], [2]. For instance, grazing can be used to modify habitat for species conservation, as demonstrated through improvements of grassland bird habitat [10], [11].

Understanding the role of grazing in species conservation requires a thorough assessment of the various components of the grazing regime. Grazing intensity and grazer identity are key components that have received the most attention [12], [13], [14], [15]. In contrast, much less is known about how manipulations of timing of grazing can affect grassland communities. Moreover, although some studies have manipulated grazers at broad temporal scales (grazing across seasons) [16], [17], [18], very few have examined the effects of timing of grazing at short time scales (within a season) [19]. Timing of grazing management decisions, such as when to initiate grazing, may be especially pertinent for conservation practitioners interested in using grazing as an effective habitat management tool in high-elevation grasslands where grazing only occurs during a contracted growing season.

Altering timing of grazing within a short growing season may have large effects on multiple trophic levels if grazing time periods coincide with key life history stages of organisms in the ecological community (e.g., reproduction, rapid growth) [20]. Livestock can reconstruct grassland bird habitat through grazing-induced modifications to vegetation structure [11]. In this way, timing of grazing may affect higher trophic levels if grazing alters the vegetation structure, either favorably or unfavorably, during key life history phases (e.g., nesting for bird species). Timing of grazing may also affect higher trophic levels through alterations to the arthropod community. For instance, many grassland birds require large amounts of energy and protein, often acquired from arthropod prey, during chick development and adult molting [21], [22], [23]. Grassland birds may be negatively affected by reductions in the density, biomass, and diversity of arthropods with grazing [3], [14], [24] if such reductions coincide with birds' key life history stages. Similarly, grassland predators, such as spiders, may be highly sensitive to temporal changes in vegetation structure and prey availability; hence, timing of grazing may have important cascading effects on the structure and stability of invertebrate food webs [25].

In addition to uncertainties with respect to timing of grazing, little is known about how grazing regimes will affect plant and arthropod communities among different grassland habitat types that form the broader vegetation mosaic. Wet meadow and upland grassland habitats often differ in soil moisture, soil type, and major limiting resources [13], which are known to influence community structure and productivity of grassland plants [26], [27]. Moreover, the effects of grazing on plant species composition and biomass may be mitigated by favorable soil water conditions [26], [28]. Having a clearer understanding of how plant and arthropod communities respond to timing of grazing across wet meadow and upland grassland habitats will be useful for land managers and livestock producers interested in adaptive grazing management.

In an effort to quantify the effects of timing of grazing on plants and arthropods in high-elevation grasslands, we experimentally manipulated timing of cattle grazing over the course of two growing seasons in two grassland habitat types. We asked: (1) how do ungrazed wet meadow and upland grasslands differ in terms of plant, arthropod, and soil moisture characteristics, (2) how does timing of grazing affect plant and arthropod communities in upland grasslands, and (3) what is the range of possible plant and arthropod responses to timing of grazing across wet meadow and upland grasslands? We hypothesized that ungrazed wet meadow grasslands would have greater soil moisture than ungrazed upland grasslands due to the closer proximity of ground water to the soil surface in wet meadows [29]. In addition, we expected greater plant biomass and arthropod densities in wet meadows resulting from the increased soil moisture. While we expected that grazing in upland grasslands would temporarily reduce graminoid and forb biomass and vegetation height, we hypothesized that plots grazed earlier in the growing season would have greater plant biomass and height towards the end of the growing season than plots grazed later in the growing season due to the increased time available for regrowth [30]. Additionally, we hypothesized that arthropod orders (i.e., Hemiptera [true bugs] and Araneae [spiders]) strongly affected by vegetation structure [3], [31], [32] would respond to grazing in upland grasslands in a similar manner as plants. Finally, we hypothesized that there would be a range of outcomes for plant and arthropod responses to timing of grazing across grassland habitat types. In particular, we expected reduced effects of grazing in wet meadow grasslands versus in upland grasslands due to increased soil moisture and plant regrowth potential [26], [17]. Our results provide a more comprehensive understanding of the effects of timing of grazing on plant and arthropod communities, as well as how these effects may differ depending on grassland habitat type. This knowledge is important for understanding how multiple trophic levels are affected by timing of grazing through temporal shifts in vegetation structure and forage availability. Our results are also highly relevant for the conservation and management of high-elevation grasslands that have extremely short growing seasons relative to lower elevations.

## Methods

### Ethics Statement

This field study was conducted in collaboration with the US Fish and Wildlife Service and The Nature Conservancy; all permissions for site access were granted and no permits were required. This study did not involve any endangered or protected species.

### Study Area

We conducted grazing experiments in 2011 and 2012 in the Centennial Valley of southwest Montana (44°40′ N, 111°47′ W, 2030 m elevation) on grasslands leased or owned by Red Rock Lakes National Wildlife Refuge (RRL) and The Nature Conservancy. Climate in the Centennial Valley is characterized by long, cold winters and short, mild summers with highly variable annual precipitation [29]. Mean annual air temperature and precipitation at Lakeview, Montana (located in the southern region of the Centennial Valley; c. 11 km from study sites) are 1.56°C and 500 mm, respectively, with May and June typically being the wettest months [29].

Our 2011 grazing experiment was conducted in wet meadow grasslands, while our 2012 experiment took place in upland grasslands. Wet meadow grasslands occupy over 2,800 hectares at RRL, and vegetation in this habitat is dominated by a dense layer of graminoids and low forb canopy cover [29]. Dominant graminoids include tufted hairgrass (*Deschampsia cespitosa*), clustered field sedge (*Carex praegracilis*), and mat muhly (*Muhlenbergia richardsonis*), while Baltic rush (*Juncus balticus*) is found in wetter areas [29]. Common wet meadow forbs include Rocky Mountain iris (*Iris missouriensis*), common dandelion (*Taraxacum officinale*), and darkthroat shooting star (*Dodecatheon pulchellum*), among others [29]. Upland grasslands occupy more than 4,900 hectares at RRL, and are largely dominated by graminoids, such as tufted hairgrass (*Deschampsia cespitosa*), clustered field sedge (*Carex praegracilis*), basin wildrye (*Leymus cinereus*), Kentucky bluegrass (*Poa pratensis*) and Sandberg bluegrass (*Poa secunda*) [29]. Forb coverage and diversity varies in upland grasslands, depending on soil moisture and type, but silvery lupine (*Lupinus argenteus*), rosy pussytoes (*Antennaria rosea*), and common yarrow (*Achillea millefolium*) are the most common [29]. Both grassland habitat types support diverse breeding bird communities, including long-billed curlew (*Numenius americanus*), sandhill crane (*Grus canadensis*), savannah sparrow (*Passerculus sandwichensis*), western-meadowlark (*Sturnella neglecta*), and other migratory birds [29].

Current management allows grazing to occur on many of the grasslands owned or leased by RRL, with grazing intensities ranging from 0.31 to 0.85 AUM/acre (where 1 AUM = 1 cow-calf pair per month) between 1994 and 2006 (U.S. Fish and Wildlife Service *unpubl. data*). Additionally, since the 1950s, 90% of grazing has been initiated after July 10^th^ due to concern over nest trampling of protected avian species (e.g. long-billed curlew and sandhill cranes). Although the long-term grazing history of the specific sites used in this study was unavailable because they were privately owned until 2008, most grazing units (ranging in size from 25 to 3,000 acres) owned or leased by RRL are grazed by cattle on a 3-year rest-rotation [29].

### Experimental Design

In both years, we established two cattle grazing treatments that differed in grazing initiation dates: early graze (starting mid-June) and traditional late graze (starting mid-July). We also established ungrazed, control plots (hereafter, ungrazed plots refers to these plots which were not grazed in the study year growing season, but may have been grazed prior to the three year rest-rotation). In 2011, our grazing experiment was conducted at a large spatial scale, using c. 38 hectare (∼1120 m by 340 m) experimental plots in heterogeneous, sub-irrigated wet meadow grasslands. We observed a high degree of patchiness in grazing perhaps due to the large amount of heterogeneity in plant community structure and soil moisture in wet meadows. In 2012, we used smaller experimental plots (c. 2 hectare, ∼135 m by 150 m) in nearby homogeneous, non-irrigated upland grasslands. Experimental blocks containing control, early-graze, and late-graze plots were established in both years. We had three blocks in 2011, with each block sized ∼114 hectares (∼1120 m by 1016 m), and four blocks in 2012, each block sized ∼6 hectares (∼405 m by 150 m). Within each block, plots were randomly selected for one of the three treatments. Due to trespass grazing in early July of 2012, two plots were removed from analyses (one control and one early-grazed), thus reducing the number of replicates for control and early-grazed treatments from four to three. In both years, early- and late-graze treatment plots were grazed by cattle for equal durations (grazed for 2 weeks) and at equal intensities (0.9 AUM/acre).

Vegetation and arthropods were collected concurrently within each plot at six sampling events between mid-June and early September in 2011 and four sampling events between early June and late August in 2012. Sampling event timing and number of events differed between years due to a shorter growing season in 2012 than in 2011. On each sampling event in 2011, ten sampling points were randomly selected for sampling out of 25 sampling points randomly established in each plot. On each sampling event in 2012, ten sampling points that were randomly established east or west of a centrally located north-south transect within each plot were sampled. Since cattle severely trampled the area near water tanks, we created a 30 m buffer zone near the water tanks where no sampling occurred. In both years, we avoided potential “edge effects” between treatments by not sampling in 10 m buffer zones between neighboring plots.

### Vegetation Sampling

At each sampling point (N = 10 per plot), above-ground plant biomass was quantified by clipping vegetation to ground level in a 0.03 m^2^ quadrat in 2011 and to 5 cm in a 0.25 m^2^ quadrat in 2012. On each sampling event, we sampled in different ordinal directions from sampling points to avoid clipping the same vegetation. Quadrats were located at least 1 m away from previously sampled quadrats. Total plant biomass included both live and dead vegetation that represented the previous and current year of growth, with dead vegetation being included because it represents an important structural habitat for arthropods and birds [31], [33], [34]. Vegetation samples were sorted as graminoids or forbs. In order to further examine grazing effects on vegetation structure, we also sampled vegetation height in 2012. Thus, prior to clipping vegetation, we measured plant height on three out of the four sampling events by estimating the height of 80% of vegetation at four equally-spaced points within the 0.25 m^2^ quadrat [35].

### Arthropod Sampling

In both years, prior to sampling vegetation at each sampling point, we sampled arthropods using an enclosed 0.25 m^2^ plastic barrel that was quickly placed over the collection area, minimizing arthropod escape and restricting the sample to a known area. We then used an inverted leaf blower/vacuum sampler (suction cylinder area of 0.013 m^2^; Craftsman XRZ 2000; 30s/sample) to extract arthropods by moving the sampler evenly across the vegetation in the enclosed area [36]. In 2012, we also used sweep netting in an effort to sample a larger area of the plot. Sweep netting took place during hours of peak arthropod activity (11:00–15:00) on calm, sunny days. We collected a total of 36 sweep net samples on sampling events 2–4 by walking swiftly in the center of each plot in a north-south transect for 50 m (40 sweeps per sample). All arthropods from each sample were frozen until processed. Arthropod density and biomass were determined for a randomly selected set of five out of ten sampling points per plot in 2011 and for all ten sampling points per plot in 2012. In both years, density was determined by separating arthropods greater than 1 mm from vegetation debris, counting individuals, and then identifying individuals to order and size class. Size classes varied depending on the arthropod order in 2011, and in 2012 all arthropod orders were measured to the nearest mm. Biomass was determined for the six arthropod orders that collectively made up over 95% of total composition in terms of abundance (Hemiptera [true bugs], Araneae [spiders], Hymenoptera [mainly ants], Diptera [flies], Coleoptera [beetles], and Orthoptera [grasshoppers]). Total arthropod biomass refers to the sum of these six arthropod orders. In 2011, we dried and weighed arthropods in each size class to obtain dry mass. In 2012, we used more specific size classes (to the nearest mm) and converted the length of each arthropod to dry mass using taxon-specific length/mass regression equations [37]. Total biomass for each arthropod order was calculated as the sum of all size classes in each sample. Individual biomass (a measure of average size) was calculated as total biomass/number of individuals for each sample.

### Soil Moisture Sampling

We measured soil moisture in each grassland habitat type to determine how local-scale water availability influenced plant and arthropod responses to grazing treatments. In the wet meadow grassland (2011), we measured soil moisture at each of the 25 sampling points in each plot three times throughout the summer. In the upland grassland (2012), we measured soil moisture at each of the ten sampling points in each plot on all four sampling events. Three soil moisture readings were taken within 30 cm of each other using an Aquaterr 300-T soil probe at a depth of 15 cm. All readings were within 60 cm of the sampling point.

### Statistical Analyses

In the wet meadow grassland experiment (2011), we did not detect strong grazing effects because of large variability in soil moisture and plant biomass within experimental plots resulting from sub-irrigation and visible heterogeneity in cattle grazing. Additionally, the discrepancy between the large plot sizes relative to the small quadrats used for plant sampling in 2011 may not have adequately captured the mean effects of the grazing treatments. We therefore restricted our in-depth analysis of timing of grazing effects throughout the growing season to the upland grassland experiment conducted in 2012. In contrast, we used both years of data to examine the range of possible plant and arthropod responses to timing of grazing across grassland habitat types (wet meadow vs. upland grassland). By comparing cumulative (late August =  end-of-season) responses, grassland communities had a longer timeframe to develop patterns and reflect grazing treatment differences between grassland habitat types.

#### Differences Between Ungrazed Wet Meadow and Upland Grasslands

To quantify grassland habitat type differences without the added complexity of grazing, we compared plant, arthropod, and soil moisture metrics from ungrazed plots in wet meadow and upland grasslands. We focused on comparing end-of-season characteristics because this time period represented the cumulative effects over most of the growing season. We first compared mean graminoid biomass, forb biomass, total arthropod density, and total arthropod biomass in each grassland habitat type using paired t-tests.

Histograms of a cumulative soil moisture metric (range 0–200%) were generated to depict end-of-season soil moisture for each grassland habitat type. This cumulative metric, based on the summed average soil moisture values from all August sampling events, was used to better represent moisture conditions in plots at the end of the growing season. To compare soil moisture between grassland habitat type, we limited the sampling points to N = 10 by randomly subsetting ten sampling points in the wet meadow (out of 25 possible) on each sampling event to match N = 10 in the upland grassland. Due to non-normality of distributions, we conducted a Kolmogorov-Smirnov two-sample test to examine inter-habitat differences in soil moisture.

To examine relationships between plant and arthropod variables across habitat types, we calculated Pearson correlation coefficients for end-of-season plant biomass and arthropod density in each grassland habitat type. We also used Pearson correlation coefficients to examine finer-scale relationships between microsite cumulative soil moisture and end-of-season (1) graminoid biomass, (2) forb biomass, (3) total arthropod density, and (4) total arthropod biomass.

#### Timing of Grazing Effects on Plants and Arthropods Across the Growing Season in Upland Grasslands

To determine the effects of timing of grazing on plant and arthropod characteristics in the upland grassland, we used a repeated-measures analysis of variance (rmANOVA). Treatment and sampling event (sampling event as the repeated measure) were included as independent variables, as well as the interaction term between them. When significance in main effects or interactions were detected (p<0.05), *post-hoc* one-way ANOVAs and Tukey's Honestly Significant Difference (HSD) multiple comparisons were used to determine treatment differences within each sampling event, as well as sampling event differences within each grazing treatment. We did not adjust alpha levels with a Bonferroni correction because doing so may lead to a higher probability of a type II error and a lack of a standard alpha across studies [38]. All plant and arthropod variables were square-root transformed +0.5 to meet assumptions of normality and homogeneity of variances [38].

Because patterns in sweep net sample results were similar to vacuum sample results, we only report the vacuum sample results because they can be expressed on a per area basis. All data were analyzed using R version 2.15.2 [39] and JMP 10 [40].

#### Timing of Grazing Effects on End-of-Season Plants and Arthropods in Wet Meadow and Upland Grasslands

Our two study years varied in terms of grassland habitat type, but also in accumulated precipitation (averaged 460 mm in 2011 and 400 mm in 2012 [41]), timing of snowmelt (1 month earlier in 2012; data from Natural Resources Conservation Science SNOTEL site at Tepee Creek; c. 24 km from study sites [42]), and length of growing season (defined as the period of time between the last frost of spring and the first frost of fall; 86 days in 2011 and 74 in 2012; data from local MesoWest fire tower data [43]). These differences in climate, along with grassland habitat type differences, allowed us to examine the range of possible plant and arthropod responses to timing of grazing. In order to compare the magnitude and direction of timing of grazing effects on plants and arthropods in two grassland habitat types, we conducted an effect size analysis which allows a standardized (i.e., responses relative to the control) comparison of responses across multiple studies, despite differences in methodology [44]. For each timing of grazing treatment, we calculated the standardized mean difference between a given plant or arthropod variable in treatment versus control plots using mean log response ratios [45]. We examined end-of-season characteristics, corresponding to late August when plants began to die back, as well as a time when the effects of herbivory on plants are most likely to influence next year's growth [46]. We conducted this analysis on graminoid biomass, forb biomass, and density and biomass of arthropod orders that collectively made up over 95% of total abundance in each study year (Hemiptera, Araneae, Hymenoptera, Diptera, Coleoptera, and Orthoptera). For both years, we used taxon-specific length/mass regression equations [37] using mean lengths based on the larger size class ranges that we established in 2011 in order to standardize methodology in biomass calculations across years. A negative value of the log response ratio indicated lower values for the response variable in the treatment plots compared to the control plots. Effect sizes for each response variable were summarized with a random-effects model using 95% confidence intervals with bias-corrected bootstrapping [44]. The effect size was considered statistically significant if the bootstrapped confidence interval, calculated with 1000 iterations, did not bracket zero. An effect size of 0.2 is considered “small”, 0.5 is “medium”, 0.8 is “large”, and anything greater than 1 is “very large” [45]. All effect size calculations were conducted using MetaWin version 2.1 [44].

## Results

### Differences Between Ungrazed Wet Meadow and Upland Grasslands

**Figure 1:**
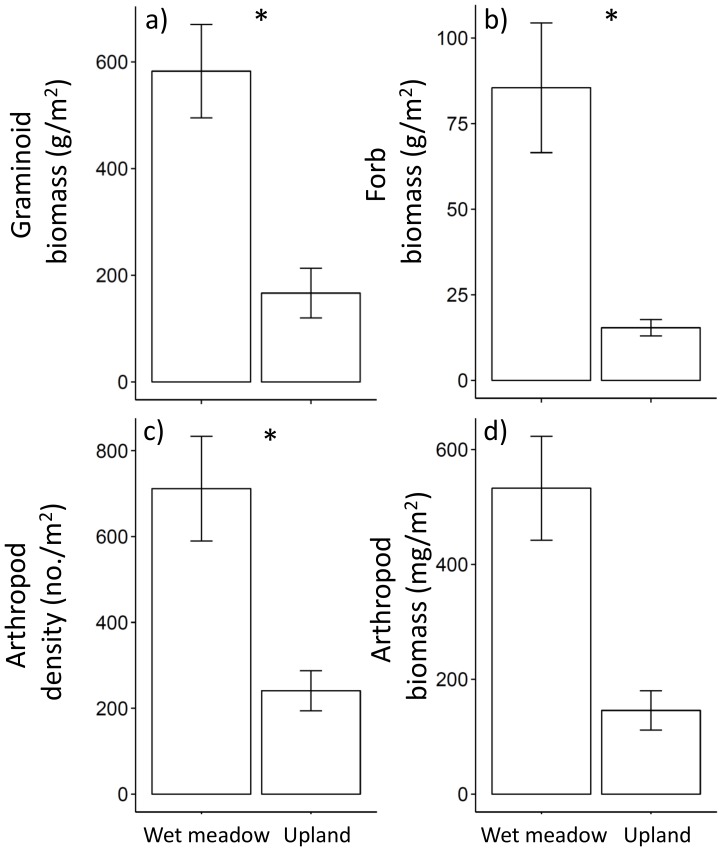
End-of-season comparisons for key grassland variables for ungrazed wet meadow and upland grasslands. End-of-season (late August) values for a) graminoid biomass, b) forb biomass, c) arthropod density, and d) arthropod biomass are untransformed means ± 1 SE. Wet meadow grasslands were sampled in 2011 and upland grasslands in 2012. * Indicates a significant difference between wet meadow and upland grasslands (p<0.05 from paired t-tests, n = 3). All variables were square-root transformed +0.5 for t-tests.

**Figure 2:**
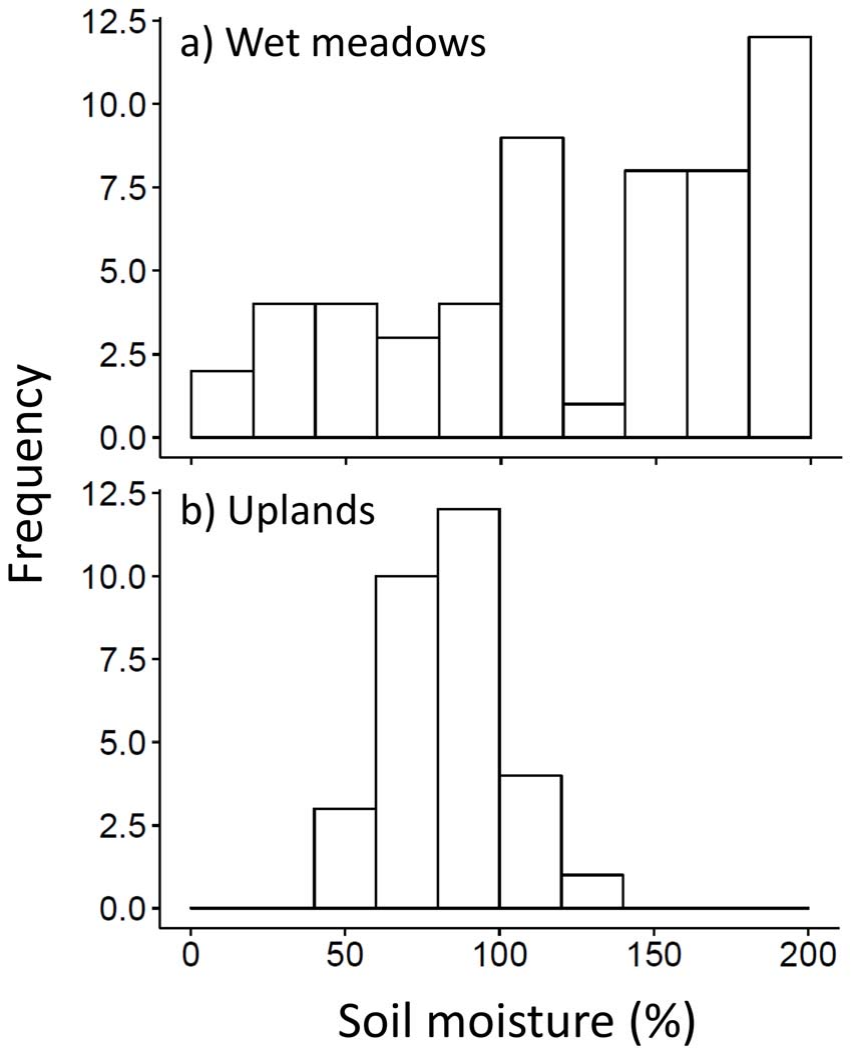
Histograms for end-of-season cumulative soil moisture according to ungrazed grassland habitat type: a) wet meadows and b) uplands. Wet meadow grasslands were sampled in 2011 and upland grasslands in 2012.

At the end of the season, ungrazed wet meadow and upland grassland habitats differed substantially in many plant and arthropod characteristics. The wet meadow grassland had greater graminoid and forb biomass, as well as total arthropod density (Figure 1a, b, c). In contrast, there were no inter-habitat differences in total arthropod biomass (Figure 1d). Soil moisture differed significantly between grassland habitat types (Kolmogorov-Smirnov test; p<0.05; Figure 2) with greater cumulative soil moisture in wet meadows (mean of 116% and median of 115%) versus uplands (mean of 81% and median of 82%; Figure 2). Total arthropod density was positively correlated with both graminoid (r = 0.06, p = 0.012) and forb biomass (r = 0.55, p = 0.03) in the wet meadow grassland. In contrast, neither graminoid nor forb biomass was significantly correlated with arthropod density in the upland grassland. Cumulative soil moisture was negatively correlated with end-of-season graminoid (r = −0.44, p = 0.02) and forb biomass (r = −0.49, p = 0.007) in ungrazed plots in the upland grassland. All other correlations were non-significant.

### Timing of Grazing Effects on Plants and Arthropods Across the Growing Season in Upland Grasslands

**Figure 3:**
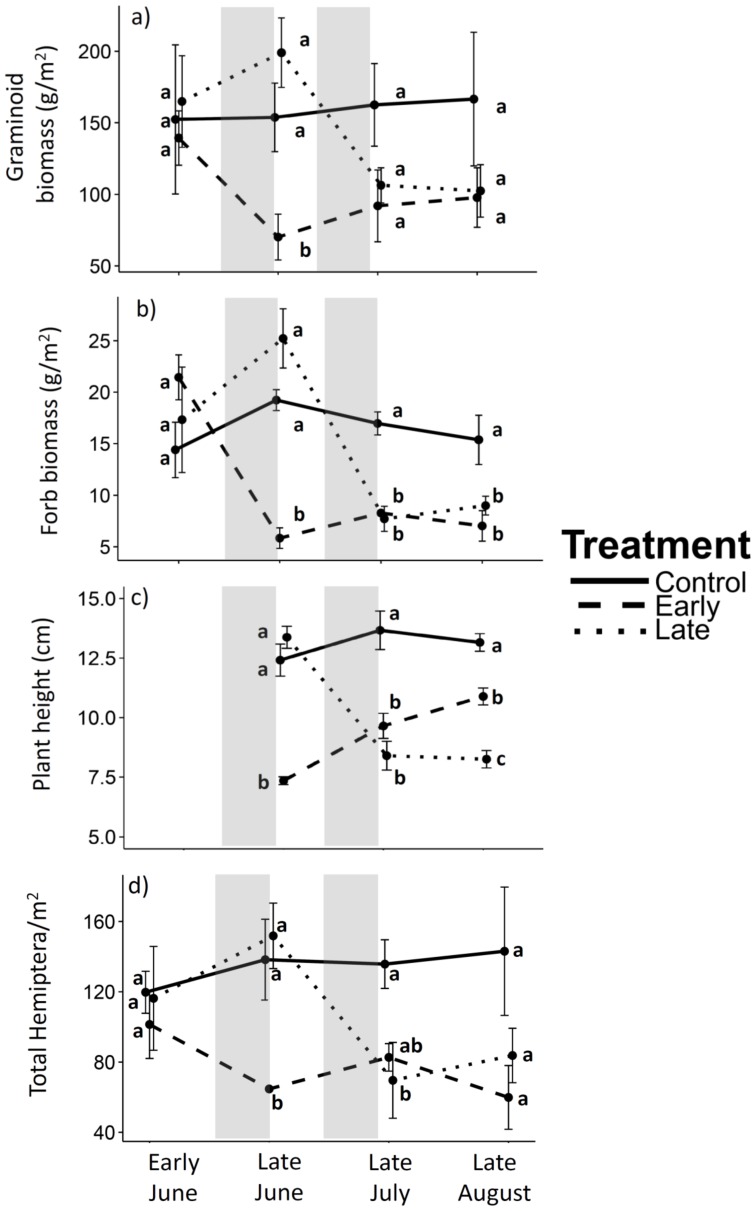
Temporal effects of grazing treatments on plant and arthropod variables for the upland grassland. Values for a) graminoid biomass, b) forb biomass, c) plant height (not sampled in early June), and d) Hemiptera density are untransformed means ± 1 SE. Duration of early and late grazing treatments are shown by shaded grey boxes. Significant treatment differences within a sampling event are indicated by differing letters. Tukey's HSD results for significant sampling event differences within a treatment are shown in Table 3.

Average graminoid and forb biomass in upland grasslands differed significantly across grazing treatments, but these effects depended on the sampling event (Table 1). Both graminoid and forb biomass were similar in all treatments prior to any grazing (Table 2) and remained constant in control plots throughout the growing season (Table 3). Forb biomass was significantly reduced by 73% (by 15.6 g/m^2^; Figure 3b; Table 3) with early grazing, whereas both graminoid and forb biomass were significantly reduced with late grazing (by 47% [by 92.67 g/m^2^] and by 69% [by 17.51 g/m^2^], respectively; Figure 3a, b; Table 3). Average graminoid biomass in both grazed treatments remained at least 30% lower than control plots throughout the rest of the growing season, although neither treatment was significantly different from control plots (Figure 3a; Table 2). The only significant difference among treatments for graminoid biomass was immediately after the early graze in late June, when early-grazed plots had less than half the graminoid biomass of the other plots (Figure 3a; Table 2). There was also a significant difference among treatments for forb biomass immediately after the early graze in late June, as well as between control plots and both early- and late-grazed plots in July and August (Figure 3b; Table 2). Forb biomass in both grazed plots was significantly lower (<50%) than control plots at the end of the growing season (Figure 3b; Table 2).

**Table 1:** Results of repeated-measures ANOVA for the effect of timing of grazing on plant and arthropod variables in upland grasslands (2012).

Variable	df_N_, df_D_	F	p-value	Arthropod group density	df_N_, df_D_	F	p-value
*Graminoid biomass*				*Hemiptera density*			
Treatment	2,7	1.86	0.2251	Treatment	2,6	3.18	0.1143
Event	3,5	3.67	0.0977	Event	3,4	9.28	**0.0283**
Treatment:Event	6,10	6.88	**0.0041**	Treatment:Event	6,8	5.92	**0.0125**
*Forb biomass*				*Hymenoptera density*			
Treatment	2,7	7.87	**0.0162**	Treatment	2,6	0.91	0.4522
Event	3,5	7.2	**0.0291**	Event	3,4	0.75	0.5763
Treatment:Event	6,10	9.74	**0.0011**	Treatment:Event	6,8	0.43	0.8376
*Plant height*				*Araneae density*			
Treatment	2,7	31.53	**0.0003**	Treatment	2,6	0.04	0.9653
Event	2,6	0.74	0.5200	Event	3,4	7.45	**0.0409**
Treatment:Event	4,12	21.40	**<0.0001**	Treatment:Event	6,8	1.17	0.4078
*Arthropod density*				*Coleoptera density*			
Treatment	2,6	2.31	0.1805	Treatment	2,6	0.34	0.7232
Event	3,4	1.65	0.3128	Event	3,4	34.87	**0.0025**
Treatment:Event	6,8	1.75	0.2277	Treatment:Event	6,8	1.70	0.2390
*Total arthropod biomass*				*Diptera density*			
Treatment	2,6	0.18	0.8417	Treatment	2,6	1.06	0.4024
Event	3,4	0.76	0.5732	Event	3,4	12.98	**0.0157**
Treatment:Event	6,8	1.09	0.4432	Treatment:Event	6,8	3.31	0.0609
*Individual arthropod biomass*				*Orthoptera density*			
Treatment	2,6	4.47	0.0647	Treatment	2,7	1.49	0.2989
Event	3,4	11.91	**0.0184**	Event	3,4	1.62	0.3192
Treatment:Event	6,8	0.73	0.6393	Treatment:Event	6,8	1.79	0.2188

Arthropod group total biomass	df_N_, df_D_	F	p-value	Arthropod group individual biomass	df_N_, df_D_	F	p-value
*Hemiptera total biomass*				*Hemiptera ind biomass*			
Treatment	2,6	0.98	0.4284	Treatment	2,6	0.29	0.7549
Event	3,4	1.26	0.3993	Event	3,4	4.18	0.1004
Treatment:Event	6,8	3.12	0.0700	Treatment:Event	6,8	1.54	0.2800
*Hymenoptera total biomass*				*Hymenoptera ind biomass*			
Treatment	2,6	0.49	0.6344	Treatment	2,6	0.85	0.4721
Event	3,4	1.58	0.3263	Event	3,4	83.93	**0.0005**
Treatment:Event	6,8	0.39	0.8639	Treatment:Event	6,8	2.13	0.1588
*Araneae total biomass*				*Araneae ind biomass*			
Treatment	2,6	0.45	0.6565	Treatment	2,6	0.24	0.7971
Event	3,4	11.05	**0.0209**	Event	3,4	1.87	0.2756
Treatment:Event	6,8	2.47	0.1176	Treatment:Event	6,8	0.93	0.5206
*Coleoptera total biomass*				*Coleoptera ind biomass*			
Treatment	2,6	0.17	0.8465	Treatment	2,6	0.37	0.7051
Event	3,4	8.01	**0.0363**	Event	3,4	0.51	0.6941
Treatment:Event	6,8	1.42	0.3159	Treatment:Event	6,8	0.94	0.5177
*Diptera total biomass*				*Diptera ind biomass*			
Treatment	2,6	0.45	0.6565	Treatment	2,6	0.24	0.7971
Event	3,4	11.05	**0.0209**	Event	3,4	1.87	0.2756
Treatment:Event	6,8	2.47	0.1176	Treatment:Event	6,8	0.93	0.5206
*Orthoptera total biomass*				*Orthoptera ind biomass*			
Treatment	2,6	1.13	0.3824	Treatment	2,6	0.92	0.4476
Event	3,4	13.50	**0.0147**	Event	3,4	38.33	**0.0021**
Treatment:Event	6,8	0.53	0.7722	Treatment:Event	6,8	3.83	**0.0422**

Sampling event is the repeated measure. Significant p-values at α<0.05 are in bold; df = degree of freedom.

**Table 2:** Univariate one-way ANOVAs for each sampling event and Tukey's Honestly Significant Difference multiple comparisons when a significant treatment effect was present within each sampling event for upland grasslands (2012).

Variable	Sampling event	df_N_, df_D_	F	p-value	Tukey's
*Graminoid biomass*					
	Early June	2,7	0.10	0.9067	
	Late June	2,7	14.03	**0.0036**	C = L
	Late July	2,7	3.01	0.1141	
	Late August	2,7	2.02	0.2032	
					
*Forb biomass*					
	Early June	2,7	0.87	0.4596	
	Late June	2,7	29.23	**0.0004**	C = L
	Late July	2,7	22.68	**0.0009**	E = L
	Late August	2,7	8.67	**0.0128**	E = L
					
*Plant height*					
	Late June	2,7	54.18	**<.001**	C = L
	Late July	2,7	16.37	**0.0023**	E = L
	Late August	2,7	40.95	**0.0001**	
					
*Hemiptera density*					
	Early June	2,7	0.26	0.7795	
	Late June	2,7	11.02	**0.0098**	C = L
	Late July	2,7	6.53	**0.0251**	C = E, E = L
	Late August	2,7	3.24	0.1008	
					
*Hemiptera total biomass*					
	Early June	2,7	0.98	0.4231	
	Late June	2,7	0.97	0.4302	
	Late July	2,7	3.52	0.0874	
	Late August	2,7	2.13	0.1891	
					
*Orthoptera ind biomass*					
	Early June	2,7	5.68	**0.0342**	C = E, E = L
					
	Late June	2,7	1.08	0.3965	
	Late July	2,7	0.30	0.7511	
	Late August	2,7	0.06	0.9418	

Significant p-values at α<0.05 are in bold; df = degree of freedom. The treatment letters sharing a line are not statistically different from one another. Treatments are indicated as follows: C = Control, E = Early, L = Late.

**Table 3:** Univariate one-way ANOVAs for each treatment and Tukey's Honestly Significant Difference multiple comparisons when a significant sampling event effect was present within each treatment for upland grasslands (2012).

Variable	Treatment	df_N_, df_D_	F	p-value	Tukey's
*Graminoid biomass*					
	Control	3,8	0.06	0.9829	
	Early	3,8	2.86	0.1044	
	Late	3,8	4.81	**0.0200**	1 = 2, 1 = 3 = 4
					
*Forb biomass*					
	Control	3,8	1.31	0.3366	
	Early	3,8	22.22	**0.0003**	2 = 3 = 4
	Late	3,8	9.49	**0.0017**	1 = 4, 3 = 4
					
*Plant height*					
	Control	2,6	0.93	0.4458	
	Early	2,6	26.35	**0.0011**	3 = 4
	Late	2,6	30.60	**<0.0001**	3 = 4
					
*Hemiptera density*					
	Control	3,8	0.20	0.8967	
	Early	3,8	1.62	0.2598	
	Late	3,8	4.41	**0.0288**	1 = 2 = 4, 1 = 3 = 4
					
*Hemiptera total biomass*					
	Control	3,8	0.98	0.4486	
	Early	3,8	5.18	**0.0279**	1 = 2 = 4, 1 = 3 = 4
	Late	3,8	1.26	0.336	
					
*Araneae density*	NA	3,8	6.75	**0.001**	1 = 4, 2 = 3 = 4
					
*Coleoptera density*	NA	3,8	12.72	**<0.0001**	2 = 3 = 4
					
*Diptera density*	NA	3,8	17.30	**<0.0001**	2 = 3 = 4
					
*Hymenoptera individual biomass*	NA	3,8	6.99	**0.0008**	1 = 2 = 3, 3 = 4
					
*Araneae total biomass*	NA	3,8	9.84	**<0.0001**	2 = 3 = 4
					
*Coleoptera total biomass*	NA	3,8	7.35	**0.0006**	2 = 3 = 4
					
*Diptera total biomass*	NA	3,8	9.84	**<0.0001**	2 = 3 = 4
					
*Orthoptera total biomass*	NA	3,8	5.39	**0.0037**	1 = 2, 2 = 3 = 4
					
*Orthoptera individual biomass*					
	Control	3,8	1.09	0.4075	
	Early	3,8	6.21	**0.0175**	1 = 2 = 3, 2 = 3 = 4
	Late	3,8	2.64	0.1019	
					
*Individual arthropod biomass*	NA	3,8	3.85	**0.0176**	1 = 2 = 4, 2 = 3 = 4

Significant p-values at α<0.05 are in bold; df = degree of freedom. The sampling event numbers sharing a line are not statistically different from one another. Sampling events are indicated as follows: 1 = Sampling event 1 (early June), 2 = Sampling event 2 (late June), 3 =  Sampling event 3 (late July), 4 =  Sampling event 4 (late August). If arthropod orders did not vary according to treatment, analysis was done on means across all treatments (variables where treatment = NA).

Average plant height differed significantly across grazing treatments, but these effects depended on the sampling event (Table 1). We assumed there were no pre-treatment differences in plant height because graminoid biomass was similar in all treatments prior to any grazing (Table 1) and post-treatment plant height was strongly correlated with graminoid biomass (r = 0.72, p<0.0001, Pearson's correlation). Plant height responses to grazing were similar to graminoid and forb biomass responses, but plant height differed significantly between early- and late-grazed plots at the end of the growing season (Figure 3c; Table 2). Although early- and late-grazed plots had equal graminoid biomass at the end of the growing season (Figure 3a; Table 2), early-grazed plots had 17% taller vegetation compared to late-grazed plots in late August (Figure 3c; Table 2).

Arthropod density in control plots was largely composed of Hemiptera (57%), Hymenoptera (23%), and Araneae (10%) across all sampling events. The remaining 10% was composed of Coleoptera, Diptera, Orthoptera and six additional low-density orders. Patterns with respect to total arthropod biomass were similar, with the majority of arthropod biomass consisting of Hemiptera (40%), Hymenoptera (19%), Araneae (16%), and Orthoptera (14%).

Average Hemiptera density differed significantly across grazing treatments, but these effects depended on the sampling event (Table 1). Hemiptera density was similar in all treatments prior to any grazing (Table 2) and remained constant in both control and early-grazed plots throughout the growing season (Table 3). Late grazing significantly reduced Hemiptera density by 54% (by ∼82 individuals/m^2^; Figure 3d; Table 3). Early- and late-grazed plots differed significantly immediately after the early graze in late June, when early-grazed plots had less than half the density of Hemiptera than the other treatments (Figure 3d; Table 2). At the end of the growing season, Hemiptera density in grazed plots did not differ from control plots (Figure 3d; Table 2).

There was no effect of grazing on average body size (i.e., mean individual biomass) of Hemiptera. However, there was a marginally significant effect of grazing on Hemiptera total biomass (p = 0.07; Table 1), but this effect was only evident in late July; late-grazed plots had 37% less Hemiptera biomass than control plots, but only 6% less Hemiptera biomass when compared to early-grazed plots (Table 2). In early June, prior to any grazing, control plots had larger Orthoptera than late-grazed plots (Table 1, Table 3). Grazing did not significantly affect the density or biomass of any other arthropods (Table 1).

### Timing of Grazing Effects on End-of-Season Plants and Arthropods in Wet Meadow and Upland Grasslands

**Figure 4:**
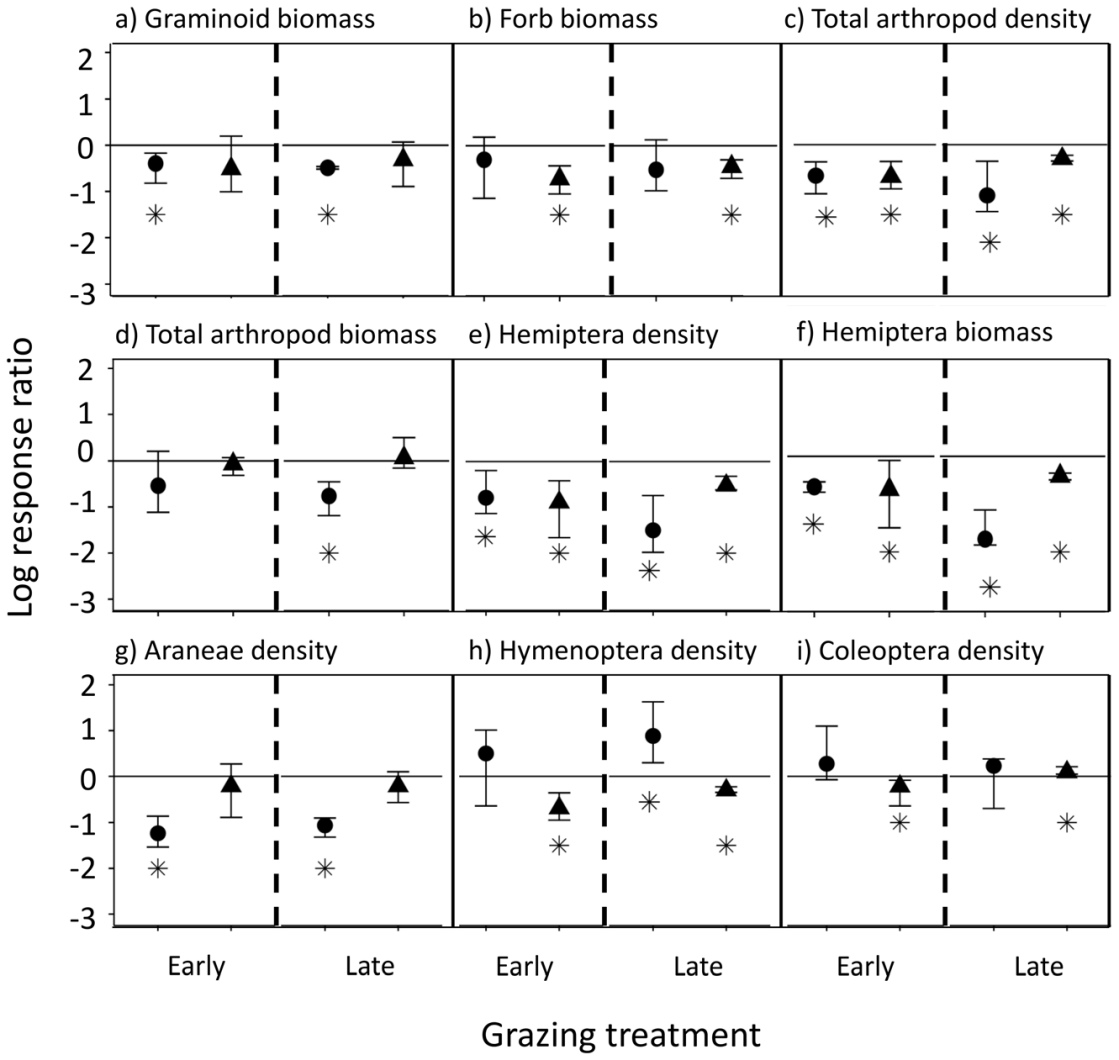
Effect sizes (log response ratio) for end-of-season plant and arthropod variables in wet meadow and upland grasslands. Wet meadows are represented with a circle and uplands with a triangle. The log response ratio compares grazing treatments to control and asterisks (*) denote significant effect sizes at α = 0.05. Error bars are bias-corrected 95% confidence intervals. Due to similar results for density and biomass for Araneae, Hymenoptera, and Coleoptera, we only report effect sizes for arthropod density. Diptera and Orthoptera density and biomass in grazed treatments were not significantly different from controls.

Effect sizes of grazing treatments on end-of-season graminoid biomass and forb biomass varied depending on the grassland habitat type. Regardless of timing, grazing had a medium negative effect on end-of-season graminoid biomass in the wet meadow grassland and forb biomass in the upland grassland (Figure 4a, b). Grazing also had a medium to large negative effect on end-of-season arthropod density (driven largely by Hemiptera; Figure 4c and e) and Hemiptera total biomass in both grassland habitats (Figure 4f). Late grazing had a particularly large negative effect (−1.7) on Hemiptera total biomass in the wet meadow grassland (Figure 4f), as well as end-of-season total arthropod biomass (−1.1; Figure 4d). Regardless of timing, grazing had a very large negative effect (early = −1.1, late = −1.2) on end-of-season Araneae density in the wet meadow grassland (Figure 4g). The effects of timing of grazing on density of other taxa (e.g., Hymenoptera and Coleoptera) were more complex and idiosyncratic, with medium to large positive effects on Hymenoptera density in the wet meadow grassland and variable effects in the upland grassland (Figure 4h, i).

## Discussion

Our grazing experiment in high-elevation, western U.S. grasslands demonstrated that grazing altered plant and arthropod characteristics within a single growing season. In upland grasslands, we found that both timing of grazing treatments generally reduced plant biomass and height, and grazing earlier versus later in the growing season did not result in greater recovery for graminoid or forb biomass. Additionally, the density of the most dominant arthropod order (Hemiptera) was reduced by both grazing treatments. While both grazing initiation dates shared similarities in responses, our results suggest that early grazing has the potential to negatively affect grassland birds that are reliant on forbs and Hemiptera as a main forage source in June. Our results also suggest that the influence of grazers on plant communities may depend on both grassland habitat type and plant functional group, but climatic variation is likely to influence grazing outcomes.

### Differences Between Ungrazed Wet Meadow and Upland Grasslands

We found that the ungrazed wet meadow grassland had greater graminoid and forb biomass, as well as total arthropod density, than the ungrazed upland grassland. We expected such differences due to the influence of soil moisture in wet meadows resulting from spring flooding and closer proximity to ground water [29]. Our cumulative end-of-season soil moisture readings suggested that the wet meadow still had greater soil moisture values than the upland grassland at the end of the growing season. However, differences in plant and arthropod communities in varying grassland habitat types may also be due to interannual variation in precipitation. Our study in upland grasslands occurred during a relatively drier year compared to the year we sampled wet meadow grasslands; therefore, precipitation/soil moisture differences between grassland habitat types are likely to represent a wider range of responses than if the two study years had very similar precipitation patterns. Regardless of whether or not soil moisture differences are a result of grassland habitat type or precipitation differences, such soil moisture differences in the broader vegetation mosaic are likely to affect how grazing management between grassland habitat types impacts multiple trophic levels [13]. For instance, total arthropod density was strongly linked to graminoid and forb biomass in wet meadow grasslands, but not in upland grasslands, suggesting that grazing effects on the plant community are likely to indirectly affect the arthropod community in wet meadows.

### Effects of Timing of Grazing on Plants and Arthropods Across the Growing Season in Upland Grasslands

Many plant characteristics were temporarily reduced by both grazing treatments, as expected. However, plant height, but not plant biomass, was greater with early grazing than late grazing at the end of the season. Several explanations may help elucidate why plant biomass did not recover to a greater degree with early grazing than late grazing, despite the increased time available for regrowth. First, a plant's ability to regrow after grazing can vary with local conditions, such as the availability of nutrients and moisture [47]. Plant regrowth after grazing may have been limited in the upland grassland because precipitation in 2012 was minimal (47 mm) between June and August, the key growing season in this system. This hypothesis is further supported by the lack of plant growth in control plots across the growing season, suggesting water-limited conditions. Furthermore, the recovery of plant biomass may depend on the phenological stage of plants during grazing. Clipping studies indicate that grasses and forbs are harmed most during the transition from flower stalk formation to seed ripening, which is a time when plants are storing carbohydrates for the dormant period [48]. In our study, it is possible that the majority of grasses and forbs were in this developmental stage during grazing; however, we did not closely track phenological stages of plant species. Finally, there may have been belowground recovery from early grazing that we did not measure; grazers in Yellowstone National Park stimulated root production seven times more than shoot production [49].

We found no significant differences in graminoid biomass at the end of the season between early and late grazing, as well as between grazed and control plots. However, the 40% reduction in mean graminoid biomass in both early- and late-grazed plots (Figure 3a) suggests that graminoid biomass never fully recovered from either grazing treatment, a pattern we may have detected with increased replication. A power analysis using graminoid biomass results from late August indicated that six plots per treatment (instead of four) would be necessary to detect a treatment difference (i.e., at α = 0.05; power = 0.29).

Generalizations about effects of grazers on arthropods are elusive [50] due to the large variation in how different arthropod groups respond to grazing-mediated habitat effects [3], [12]. Our results showed that Hemiptera density followed a very similar pattern as the plant community to timing of grazing, as expected, whereas spider density was unaffected by grazing. One possible explanation for why spiders did not show a similar response as Hemiptera in our study is that a sufficient level of plant architectural diversity may have been maintained for spider habitat, irrespective of grazing [31]. Additionally, there may be species-specific responses of spiders to timing of grazing, potentially driven by variation in foraging strategy [19]. Even though the effects of timing of grazing on Hemiptera density were temporary (i.e., Hemiptera recovered from both grazed treatments by the end of the growing season), Hemiptera are an important part of this grassland community, comprising over 50% of arthropod density and over 30% of arthropod biomass. Morris and Lakhani [51] likewise found a time-specific response of Hemiptera to changes in the vegetation community in a lowland grassland; cutting the vegetation early in the growing season (May) reduced the abundance of Hemiptera, but the effects were short-lived.

With the exception of Hemiptera, our results showed that arthropod orders were unaffected by grazing across the growing season in upland grasslands. While some studies that manipulated grazing intensity concur with our results (e.g., [52]), many others have found a negative effect of grazing on arthropods [14], [15], [53]. These conflicting results may be due to differing habitat types, grazing intensity, grazer identity, and length of experiment. We may have observed stronger responses from the arthropod community with larger sample sizes and if our grazing treatments ran for longer than two weeks, although this short duration of grazing is typical due to the short growing season of our high-elevation study sites.

### Timing of Grazing Effects on End-of-Season Plants and Arthropods in Wet Meadow and Upland Grasslands

Regardless of timing of grazing, we found that end-of-season plant biomass was often reduced by grazing, but the effect depended on grassland habitat type and plant functional group. End-of-season forb biomass was unaffected by grazing in the wet meadow grassland, while end-of-season graminoid biomass was unaffected by grazing in the upland grassland. A possible reason for these differences in responses is likely related to the competitive abilities of the two functional groups. Mueggler found that forb growth benefited more from increased soil moisture than grass growth and suggested that forbs grazed during a wet year would be damaged less than those grazed during a dry year [48]. Our cumulative soil moisture readings indicated that the wet meadow grassland had a different distribution of soil moisture values than in the upland grassland. Additionally, our study in upland grasslands took place in 2012, which was a relatively drier year compared to the 2011 study year in wet meadow grasslands, indicating that our results represent two distinct ends of the abiotic spectrum for this system. These combinations of grassland habitat type and precipitation may have influenced grazing responses, but are representative of realistic conditions and are important factors to consider in adaptive grazing management decisions.

We found that some arthropod orders were unaffected by grazing (i.e., Diptera and Orthoptera), while grazing reduced many other end-of-season arthropod densities in both study years, regardless of timing of grazing or grassland habitat type. End-of-season Hemiptera density was reduced for both grazing treatments in both grassland habitat types, while Araneae density was reduced in the wet meadow grassland. While we did not find as many negative effects of grazing on arthropods *across* the growing season with the rmANOVA, the effect size analysis revealed there were still cumulative negative effects of grazing on several arthropod groups at the *end of the season*, which has important implications for arthropods' ability to recover from a grazing event.

Different plant and arthropod responses in 2011 vs. 2012 may be due to differences in grassland habitat type, but as previously stated, may also be due to interannual variation in climate, such as accumulated precipitation, timing of snowmelt, and length of growing season. It is also possible there were other unmeasured ecological drivers that varied between our two study sites and contributed to the observed responses of plants and arthropods to grazing, such as differences in fine-scale topography [26] or variability in timing of rainfall [16]. Given that the wet meadow grassland was sampled in a wet year and the upland grassland experiment in a dry year, the observed differences between grassland habitat types may be exaggerated compared to other years. Therefore, the effects of timing of grazing on plants and arthropods in upland grasslands remain elusive during a wet year, in which additional soil moisture may benefit an otherwise drier grassland habitat type. Additional studies of wet meadow and upland grasslands in alternative climatic conditions would help to refine grazing management recommendations for these grassland habitat types under variable climate.

### Management Implications

Our results demonstrate that the effects of timing of grazing on plant and arthropod communities are not uniform throughout the growing season and such temporal shifts have important implications for species conservation. First, initiating grazing earlier in the growing season in upland grasslands may impact bird species of conservation concern that rely on forbs and Hemiptera as a food source in June. Other studies have similarly suggested that temporal variation in bird forage may have important implications for post-fledgling survival [20]. The temporal shifts we observed in bird forage availability (i.e., Hemiptera) are more likely to affect smaller bird species, such as the savannah sparrow, western meadowlark, and other small-bodied passerines. Larger-sized bird species (long-billed curlew and greater sage-grouse) are less likely to be affected, as they preferably forage on larger-sized arthropod orders, such as Coleoptera [23], that were largely not affected by the grazing treatments in our study. Although we did not measure bird densities as part of this study, previous research has shown that savannah sparrows have a density of 0.52 males/ha (±0.02 SE) in wet meadows at RRL [54]; hence there are higher trophic levels that are likely to be affected by changes in their forage availability as a result of timing of grazing.

Secondly, several plant community variables were affected by timing of grazing and such alterations in vegetation structure have important implications for nesting habitat for bird species of conservation concern. Early grazing in the upland grassland resulted in 17% taller plant height at the end of the season compared to late grazing. Since residual plant height persists into the next growing season, this information could be used to inform management of grassland birds that require specific vegetation heights for fulfilling life history requirements [11]. All of our grazing treatments, including controls, had less than 15 cm plant height from June to August, indicating that long-billed curlews, with habitat requirements of less than 30 cm plant height for nesting purposes [29], would nest in this area regardless of timing of grazing. However, fine-scale differences in plant height may affect rates of nest predation for other smaller, ground-nesting grassland passerines, such as savannah sparrow and western meadowlark [55].

Proper planning of grazing regimes may aid in reducing possible negative effects of early season grazing on higher trophic levels. Early season grazing could take place outside of core bird nesting and foraging areas in the future, which may help mediate potential early-season impacts on higher trophic levels. One of the most beneficial grazing strategies may include a matrix of grazing plots, with varying grazing initiation dates and years of rest between grazing periods at the landscape level. Doing so would aid certain areas of the grassland in recovery from grazing at any point during the growing season, and provide the necessary structure and forage availability for a broad suite of species with differing life history requirements. A better understanding of how timing of grazing affects plants and arthropods will be useful for conservation grazing management in similar high-elevation grassland systems and in particular, for determining how potential changes in forage availability will affect species conservation.
